# Variation in UK fracture liaison service consultation conduct and content before and during the COVID pandemic: results from the iFraP-D UK survey

**DOI:** 10.1007/s11657-023-01361-4

**Published:** 2023-12-20

**Authors:** Laurna Bullock, Sittana Abdelmagid, Jane Fleming, Sarah Leyland, Emma M. Clark, Christopher Gidlow, Cynthia P. Iglesias-Urrutia, Terence W. O’Neill, Christian Mallen, Clare Jinks, Zoe Paskins

**Affiliations:** 1 School of Medicine, Keele University, Staffordshire, UK; 2 Guy’s and St Thomas’ NHS Foundation Trust, London, UK; 3 Cambridge Public Health, University of Cambridge, Cambridge, UK; 4 Addenbrooke’s Hospital Fracture Liaison Service, Cambridge University Hospitals NHS Trust, Cambridge, UK; 5 Royal Osteoporosis Society, Bath, UK; 6 Bristol Medical School, Faculty of Health Sciences, University of Bristol, Bristol, UK; 7 Centre for Health and Development, Staffordshire University, Stoke-On-Trent, Staffordshire UK; 8 Department of Health Sciences, University of York, York, UK; 9 Danish Centre for Healthcare Improvements (CHI), Aalborg University, Aalborg, Denmark; 10 Centre for Epidemiology Versus Arthritis, University of Manchester, Manchester, UK; 11 NIHR Manchester Biomedical Research Centre, Manchester University NHS Foundation Trust, Manchester Academic Health Sciences Centre, Manchester, UK; 12 Haywood Academic Rheumatology Centre, Staffordshire and Stoke-On-Trent Partnership Trust, Stoke-On-Trent, Staffordshire UK

**Keywords:** Fracture liaison service, iFraP, Survey, Osteoporosis, Shared decision-making

## Abstract

***Summary*:**

We conducted a survey of FLSs’ consultation conduct and content which identified marked variation in whether FLS HCPs discussed osteoporosis medicine with patients. A review of service pro formas showed more content related to ‘investigating’ and ‘intervening’ than to ‘informing’. We propose an expanded FLS typology and model FLS pro forma.

**Purpose:**

To investigate the nature of direct patient contact in fracture liaison service (FLS) delivery, examine the use and content of pro formas to guide information eliciting and sharing in FLS consultations, and determine service changes which were implemented as a result of the COVID-19 pandemic.

**Methods:**

An electronic survey of UK FLS healthcare practitioners (HCPs) was distributed through clinical networks, social media, and other professional networks. Participants were asked to upload service pro formas used to guide consultation content. Documentary analysis findings were mapped to UK FLS clinical standards.

**Results:**

Forty-seven HCPs responded, providing data on 39 UK FLSs, over half of all 74 FLSs reporting to FLS-database. Results showed variation in which HCP made clinical decisions, whether medicines were discussed with patients or not, and in prescribing practice. Services were variably affected by COVID, with most reporting a move to more remote consulting. The documentary analysis of eight service pro formas showed that these contained more content related to ‘investigating’ and ‘intervening’, with fewer pro formas prompting the clinician to offer information and support (e.g., about coping with pain). Based on our findings we propose an expanded FLS typology and have developed a model FLS pro forma.

**Conclusion:**

There is marked variation in the delivery of services and content of consultations in UK FLSs including discussion about osteoporosis medications. Clinical standards for FLSs should clarify the roles of primary and secondary HCPs and the importance of holistic approaches to patient care.

## Introduction

Fracture liaison services (FLSs) are recommended by the UK Department of Health to address secondary fracture prevention. Models of FLS vary, although key functions include the following: the systematic identification of patients with fragility fractures; assessment of bone health, and risk of falls and fracture; treatment recommendations to primary care; and follow-up to promote treatment adherence [1]. Although the FLS model of care has been shown to increase osteoporosis medication initiation and adherence rates and reduce re-fracture rates, compared to usual care [2], performance in the UK against key FLS performance indicators is variable [3]. Long-term persistence with osteoporosis medicines is particularly challenging: only 23% of patients eligible reported adhering to osteoporosis medicine 12 months after fracture [3].

Greater patient involvement in decision-making about medicines, understanding patient’s knowledge, beliefs and concerns, and providing information that is easy to understand are clinician actions which have the potential to increase patient commitment to treatment [4]. The iFraP (improving uptake of fracture prevention drug treatments) study aims to develop and evaluate a theoretically-informed, complex intervention consisting of a computerised decision support tool, clinician training package and information resources, to facilitate person-centred care and shared decision-making about osteoporosis medicine, with a long-term aim of improving informed treatment initiation and concordance [5]. Both the development work for iFraP and the subsequent trial to evaluate patient experience, clinical and cost effectiveness, are situated in FLS settings. The training package targets clinician consultation skills pertaining to person-centred care. The decision support tool is designed to be used within the clinical consultation.

Part of the key development work in designing complex interventions involves understanding how the intervention will function and interact with the context in which it is delivered [6]. Key dimensions of context include the physical, spatial, organisational, social, cultural, political, and economic features of the healthcare setting [6]. Despite international and UK standards for FLS, there remains significant variation in performance across services. In a systematic review, Ganda et al. [7] summarises international secondary osteoporosis prevention models of care into four categories. In the UK, the vast majority of FLS fall into two categories, described as ‘type A’ services that identify, investigate, and initiate treatment and ‘type B’ services that investigate patients but then refer to primary care for treatment initiation. In comparison, type ‘C’ and ‘D’ services deliver lower intensity interventions, consisting of alerting patients and primary care physicians; and patient education only. Both the UK Royal College of Physicians FLS Database (FLS-DB) [3], which audits FLSs against the Royal Osteoporosis Society (ROS) clinical standards for FLS [1], and the International Capture the Fracture programme [8] publish metrics about FLS resources and processes (such as staffing levels, investigations requested, and available interventions). However, neither the ROS clinical standards, the FLS-DB facilities audit nor the International Capture the Fracture framework recommend or explore *how* clinical information is gathered or *how* drug recommendations are made, highlighting a need to further understand sources of variation with FLS models of care.

Focus groups conducted as part of the iFraP study identified that some FLS healthcare professionals did not believe that it was their role to discuss medicines with patients. FLS healthcare professionals often did not have any direct patient contact, conducting assessments remotely and providing treatment recommendations to the GP without discussing with the patient directly. Furthermore, the COVID pandemic led to anecdotal accounts of services moving to more remote or virtual FLS assessments and interventions, reducing or changing the nature of patient contact.

As part of the development work for iFraP (iFraP-D) [5], we identified a need to understand current UK FLS practice to ensure our intervention development was fit for purpose. To address this, we undertook a survey of FLS sites across the UK. The aim of this national survey was to examine the FLS care pathway, with particular attention to the nature and extent of direct patient contact, the use and content of pro formas to guide information eliciting and sharing, and the nature and extent of service change as a result of the pandemic.

## Methods

### Survey design

The survey was designed using web-based survey software (HealthSurvey, hosted by Keele University and located on their secure servers). The content of the survey was developed in collaboration with iFraP study team members (including academics, clinicians, and an ROS representative) based on the iFraP focus group findings and informed by discussions with stakeholders and a Patient Advisory Group (PAG). The survey was piloted by three FLS healthcare professionals across two services that investigate patients but then refer to primary care for treatment initiation (type B) and updated following feedback.

### Survey content

The survey presented the participant information sheet and captured informed consent, followed by questions about which FLS the participant worked in. This included details about the service at the time of completion, including location, who it was staffed by, and the resources available to the service which might influence the ease of use of the iFraP decision support tool and/or the delivery of the trial (e.g., video consultations, facilities to email or text patients, printers). The survey comprised seven sections, each asking the participant to reflect on pre-COVID activities and patient contact at each stage of the FLS care pathway, including the following:

Step 1 Case-finding
Step 2 Clinical assessment
Step 3 Clinical decision-making about osteoporosis medicine recommendations
Step 4 Providing treatment recommendation advice to the patient
Step 5 First prescription
Step 6 3- to 4-month follow-up
Step 7 12-month follow-up

Survey participants were asked if their services used a ‘pro forma’—a document used to collect patient data in individual FLS clinical assessments, usually completed in the consultation by the clinician. If so, they were asked to upload a blank pro forma (without patient information) to the survey. Documents which contained guidance only (no sections to complete) and questionnaires to be completed by the patient were not included.

At the end of the survey, participants were asked to reflect on how each stage of the FLS care pathway for their service changed during COVID and expectations for post-COVID service provision, with a section for free text.

### Participants and recruitment

Healthcare professionals working in any UK FLS were eligible to participate. Ethical permission for the study was given by North West—Greater Manchester West Research Ethics Committee (reference number: 19/NW/0559).

Information about the study and link to access the survey was distributed by the following:

- FLS-DB and ROS national newsletters and mailing lists
- Social media advertisement
- Study team members sharing study information with individuals in their networks who were potentially eligible to participate or could facilitate onwards dissemination, including members of the British Endocrine Society, British Geriatric Society and British Society of Rheumatology. Individuals contacted by the study team by email received up to two reminders sent approximately every 10 days.

### Analysis

Survey questions asked about the service, rather than individual behaviours, meaning that duplicate entries from the same FLS were checked for consistency. If discrepancies were identified, the respondent(s) who had consented to further contact, were contacted to clarify their responses. Following clarification, duplicate entries were removed, resulting in one data entry per UK FLS. Descriptive statistical analyses were completed, including proportions and frequencies, as appropriate. Free text results were summarised narratively.

Results of the descriptive analysis were used to classify FLS types, building on the existing classification system [7] to develop a new typology.

FLS pro formas provided by participants were analysed using document analysis [9]. An extraction matrix was developed deductively using Microsoft Excel based on ROS clinical standards for FLSs which were relevant to the content of the consultation [1]. Discussions with the wider team supported updates to the extraction matrix, where appropriate, resulting in 14 possible data extraction items across three standards: standard 2 investigate, standard 3 inform, and standard 4 intervene. Standards 1 (identify), 5 (integrate), and 6 (quality) were not included as they were not relevant to the consultation content. Dual extraction was completed by two authors (SA and LB) on a subsample of pro formas (*n* = 3, 38%), where each section of each pro forma was extracted and mapped to the matrix, with interrater agreement assessed (> 90% agreement across 14 items). The remaining pro formas were scored by SA using the matrix, indicating whether for each pro forma, each item was met (data extract fully mapped to this item—present), partially met (mentioned) or not met (absent). Additional information included in pro formas, not accounted for by the ROS clinical standards, was inductively extracted verbatim.

### Patient and public involvement

Patients and public members were involved in the iFraP development studies via a dedicated PAG and as members of a mixed stakeholder (Community of Practice) group. PAG members were not involved in the design of the survey but were involved in analysis and interpretation. They outlined the importance of being involved in decisions about medicines which informed the typology and helped to advise on the implications of findings for the iFraP trial.

## Results

Survey data were collected between October 2020 and January 2021, with 47 respondents. One ineligible participant was removed (located in Republic of Ireland) giving a final sample of 46 (37 complete and 9 partial responses). The survey included data pertaining to 39 UK FLSs (32 complete responses and 7 partial responses), the majority of which were situated in secondary care (*n* = 32/39, 81%) and in England (*n* = 31/39, 79%). A summary of included FLSs, including reported resource and facility availability, is outlined in Table 1. Table 2 lists the results relating to each step of the FLS care pathway. Denominators are provided for each survey item (see Tables 1 and 2) as they varied for each question due to either partial responses or questions being conditional on previous answers.

**Table 1:** Characteristics of Included FLSs

Question	Number	Percent
Geographical location		
England	31/39	79
Scotland	3/39	8
Wales	2/39	5
Northern Ireland	3/39	8
NHS setting		
Primary care	4/39	10
Secondary care	32/39	82
Community Hospital or Division of NHS trust	3/39	8
Service staffing		
Specialist nurses	35/39	90
Pharmacists	4/39	10
GPs	2/39	5
DXA technicians or radiographers	19/39	49
Specialist doctor (geriatrician, rheumatologist)	7/39	18
Physiotherapist	5/39	13
DXA availability		
In the service	11/39	28
In the trust/organisation	23/39	59
Have to refer externally	5/39	5
Other resource and facility availability		
Face-to-face consultations	34/39	87
Telephone consultations	38/38*	100
Video consultations	17/38	45
Facility to email patients	15/38	40
Facility to text patients	7/38	18
Access to a printer	35/38	92

*DXA* dual-energy X-ray absorptiometry, *GP* general practitioner, *NHS* National Health Service

*n = 1 partial responder exits the survey lowering the total denominator

**Table 2:** Service results: pre-COVID activities

Stage 1: case-finding	Number	Percent
Any part of case finding completed face-to-face		
Yes	18/37*	49
Approximate proportion of case load identified face-to-face		
One quarter (25%)	10/18	56
Half (50%)	3/18	17
Three quarters (75%)	2/18	11
All or nearly all (~ 100%)	3/18	17
No	19/37	51
Method of informing patients of need to attend FLS if not seen face-to-face		
Telephone	17/37	46
Letter	29/37	78
Stage 2: clinical assessment	Number	Percent
Clinical assessment conducted face-to-face		
Yes		
Yes, for all	18/36*	50
Yes, for some groups	10/36	28
Length of the FLS appointment		
0–20 min	5/27	14
21–40 min	17/27	46
41–60 min	5/27	14
Availability of DXA scan results		
Performed before the clinical assessment	6/27	22
Performed same day as clinical assessment	8/27	30
Referred to DXA after clinical assessment	13/27	48
No	8/36	22
Step 3: clinical decision-making about drug treatment recommendations	Number	Percent
Who usually makes the decision about need for osteoporosis drug		
FLS healthcare professional	27/35*	77
Other non-primary care healthcare professional	5/35	14
Primary care healthcare professional (e.g. GP)	2/35	6
All of the above	1/35	3
If FLS healthcare professional, when was this decision usually made		
During the clinical assessment with patients	21/26*	81
After the clinical assessment with patients	5/26	19
Step 4: treatment recommendation advice to patient	Number	Percent
Who usually explained drug treatment recommendation first		
FLS healthcare professional	23/34	68
Other non-primary care healthcare professional	2/34	6
Primary care healthcare professional (e.g. GP)	9/34	27
If FLS healthcare professional explained the drug recommendation		
How was the recommendation given		
Face-to-face	18/23	78
Over the phone	3/23	13
In the patient’s home	1/23	4
By letter or telephone	1/23	4
How often was this given on the same day as clinical assessment		
Always or almost always	11/23	48
Often	8/23	35
Sometimes	2/23	9
Rarely	1/23	4
Never	1/23	4
Was discussion about drugs other than oral bisphosphonates…		
In the same appointment	13/23	57
In another booked appointment	10/23	44
Step 5: first prescription	Number	Percent
Who provides the first prescription		
FLS	4/34	12
GP	30/34	88
If GP provides prescription, what do the FLS advise patients to do		
To make appointment with GP	15/30	50
To collect prescription only	9/30	30
Other	6/30	20
Steps 6 and 7: follow-up	Number	Percent
Does the FLS conduct 3–4 month follow-up		
Yes	28/34	82
Face-to-face	2/28	7
Telephone	20/28	71
Letter/questionnaire	6/28	21
No	6/34	18
Does the FLS conduct 12-month follow-up		
Yes	23/34	68
Face-to-face	2/23	9
Telephone	17/23	74
Letter/questionnaire	4/23	17
No	11/34	32

*DXA* dual-energy X-ray absorptiometry, *FLS* Fracture Liaison Service, *GP* general practitioner

*n = 1 partial responder exits the survey lowering the total denominator

### Stage 1: case finding

The first stage of the FLS care pathway involves case finding; the identification of patients that require an FLS clinical assessment. Almost half of services reported identifying patients in-person (*n* = 18/37, 49%), such as in fracture clinic and inpatient ward rounds. If patients were identified remotely (e.g., by reviewing fracture clinic lists), FLSs often used multiple methods to notify patients, including telephone (46%) and letter (78%).

### Stage 2: clinical assessment

In FLSs, a clinical assessment may mean asking the patient questions to elicit fracture and fall risk factors, past medical history, and suitability for osteoporosis medicines. Half of services (*n* = 18/36) conducted all pre-COVID clinical assessment face-to-face. Notably, 10 services (28%) conducted clinical assessment face-to-face for some groups, with the reasons for face-to-face varying across these services, with some reporting face-to-face consultations for people aged 50 to 75 years old (*n* = 3), people aged over 75 (*n* = 2), people with a high risk of fracture or low bone mineral density on DXA scan (*n* = 6), those with vertebral fractures (*n* = 1), those with communication difficulties (*n* = 1) and those in an inpatient setting (*n* = 2).

### Stage 3, 4 and 5: clinical decision-making about drug treatment recommendations

Most services reported that the FLS healthcare professional makes decisions about osteoporosis medicines (*n* = 27/35, 77%), usually during the clinical assessment with the patient (*n* = 21/26, 81%).

The number of services where the FLS healthcare professional explained the medicine recommendation to the patient personally was lower (*n* = 23/34, 68%). When not completed by the FLS healthcare professional, services suggested medicine explanations were made by primary care healthcare professionals (*n* = 9/34, 27%), such as a GP.

A large proportion of FLSs (*n* = 30/34, 88%) indicated that the GP prescribes the first osteoporosis medicine (see Table 2).

### Stages 6 and 7: follow-up

Most participating services reported conducting 3–4-month follow-ups (*n* = 28/34, 82%). The number of services completing a 12-month follow-up was lower (*n* = 23/34, 68%). Telephone was the most common way of conducting follow-ups, at both 3–4 (*n* = 20/28, 71%) and 12 months (*n* = 17/23, 74%).

### Impact of COVID on service delivery

The survey also asked participants to reflect whether face-to-face contact with patients was affected during COVID at their service, and whether they envisaged that any changes to the service during this time would continue after COVID (see Table 3). Twenty-eight services reported that COVID had affected face-to-face contact with patients (*n* = 28/32, 88%), with six (19%) reporting that COVID had affected all stages of the FLS care pathway. The extent of COVID impact varied depending on the stage of the care pathway: 3–4 month and 12-month follow-ups were least impacted (*n* = 6/32, 23%, *n* = 6/23, 26%, respectively) compared with clinical assessments in which 81% (*n* = 26/32) of participating services reported pausing or changing the processes because of COVID.

**Table 3:** Survey results: Impact of COVID of service delivery

Has COVID affected the process of…	Number	Percent
Case finding		
Yes	18/32*	56
Paused	6/18	
Changes made because of COVID	12/18	
No	14/32	44
Clinical assessment		
Yes	26/32	81
Paused	7/26	
Changes made because of COVID	19/26	
No	6/32	19
Clinical decision making		
Yes	14/32	44
Paused	3/14	
Changes made because of COVID	11/14	
No	18/32	56
Giving treatment recommendations**		
Yes	12/21	57
Paused	2/12	
Changes made because of COVID	10/12	
No	9/21	43
Medication prescriptions**		
Yes	3/3	100
Paused	1/3	
Changes made because of COVID	2/3	
No	0/3	0
3–4 month follow-up**		
Yes	6/26	23
Paused	4/6	
Changes made because of COVID	2/6	
No	20/26	77
12-month follow-up**		
Yes	6/23	26
Paused	4/6	
Changes made because of COVID	2/6	
No	17/23	74
Did COVID affect face-to-face contact with patients		
Yes	28/32	88
No	4/32	13
Did COVID affect plans for face-to-face contact with patients post-COVID		
Yes	24/32	75
Unsure	4/32	13
No	4/32	13

*n = 2 partial responders exit the survey lowering the total denominator

**Only services reporting that they conducted these activities pre-COVID (see Table 2) are included. Denominators may differ because of partial responders

COVID service challenges described by participants included: difficulty accessing, or delays with investigations (bloods tests, bone density scans), dental reviews and community education; external control from hospital management on service closure/opening and redeployment of staff; difficult management decisions with less clinical information, e.g. FRAX only, without DXA or face-to-face review; reduced clinic or waiting room space; lower identification rates and increased ‘did not attend’ rates; and challenges related to technology e.g. staff learning how to use video appointments, and patients struggling to access these, or having difficulty with telephone appointments.

Positive changes were seen in being able to offer remote/virtual assessment for people, especially those living in remote areas; adapting the offer of face-to-face appointments for those who needed it most (e.g. those initiating osteoporosis drug treatment, or with hearing or visual impairment); increase presence on wards allowing for inpatient bone health assessments; and developing new leaflets and letters for patients.

Three-quarters of services (*n* = 24/32) expected service delivery changes introduced in response to COVID to continue to affect face-to-face contact with patients post-COVID.

#### Pro formas to collect clinical information

Twenty-seven (77%) services reported using a pro forma to guide the collection of clinical information during the FLS consultation. A total of ten services provided their pro forma template document, with eight anonymised services included in the document analysis as they met the necessary criteria (see Table 4).

**Table 4:**
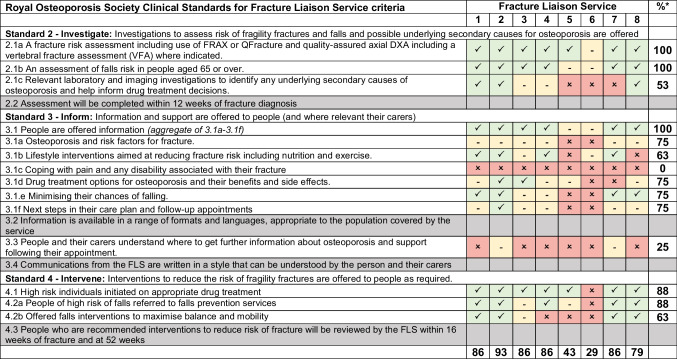
Fracture Liaison Service pro forma extraction to Royal Osteoporosis Society Clinical Standards

*Percentage (%) of items/pro formas that met or partially met the criterion. Red is not met; orange is partially met; green is met; grey cells are the items excluded as do not relate to consultation content. DXA dual-energy X-ray absorptiometry, FLS Fracture Liaison Service, GP general practitioner

Across all eight pro formas, the total number of ROS clinical standard items represented (met or partially met) ranged from 4 (29%) to 13 (93%).

Standard 2 (investigate) was well evidenced across the pro formas. All eight pro formas met (*n* = 7) or partially met (*n* = 1) criteria related to the assessment of future fracture risk and falls risk in over 65s. Fewer (*n* = 5, 53%) explicitly included reference to relevant laboratory and imaging investigations to identify any underlying secondary causes of osteoporosis.

Considering all items in standard 3 (inform), offering general additional information and support was met (*n* = 6) or partially met (*n* = 2) across all pro formas. However, pro formas rarely prompted clinicians to give information or check patient and/or carer understanding about where to access further information or support about osteoporosis following the appointment (absent in six pro formas). Although informing patients specifically about osteoporosis, risk factors for fracture and drug treatment options for osteoporosis and their benefits and side effects were represented in 75% of pro formas (*n* = 6/8), no pro forma included all of these more specific prompts. None of the pro formas prompted the clinician to ask or provide information about coping with pain and disability associated with fractures.

Items from standard 4 (intervene), such as initiation of drug treatment and falls prevention referrals at the consultation, were met or partially met in most pro formas (*n* = 7, 88%). However, pro formas less commonly prompted falls interventions to maximise balance and mobility (*n* = 5, 63%).

Inductive data extraction identified that one pro forma prompted clinicians to collect information on psychosocial factors related to social isolation, fuel poverty, and caregiver responsibilities. This same pro forma also prompted completion of the six-item Cognitive Impairment Test (6CIT). This content was not criteria listed in the ROS clinical standards.

### Typology of UK fracture liaison services

Participating services that responded to questions about osteoporosis medicine prescription (*n* = 34) were classified using a typology. Informed by previous classifications of secondary prevention care models [7], services were classified into two categories based on the service responsible for osteoporosis medicine prescription (Type A—FLS or Type B—primary care). In total, 4/34 (12%) services in this survey were classified as type ‘A’ because drug treatment was initiated as part of an all-encompassing FLS. Additional information captured in this survey provided opportunity to expand the typology further, by classifying the ‘B’ type FLSs into those who did (B1, *n* = 21/30, 70%) or did not (B2, *n* = 9/30, 30%) discuss medicines with patients.

## Discussion

We have conducted the first survey of FLSs, to our knowledge, that has focused on consultation conduct and content. We identified large variation in who makes clinical decisions, whether medicines are discussed with patients or not, in prescribing practice and whether additional GP appointments are required, or not. We identified differences also in the extent to which services were affected by the COVID pandemic, with most services reporting a move to more remote consulting. Finally, the review of service consultation pro formas suggested variation in the content of FLS consultations, with few providing detail about specific information to give to patients and none relating to symptoms or care of the index fracture.

A key driver for undertaking this survey was our previous qualitative research which found that FLS healthcare professionals did not feel it was part of their professional role to discuss medicine with patients. We found that more than a quarter of responding UK FLSs did not discuss osteoporosis medicines with patients. This is important because findings from our iFraP qualitative study indicated barriers to GPs discussing osteoporosis medicines with patients: some GPs did not feel confident, describing FLS as ‘best placed’ to have discussions about medicines, whilst other GPs reflected on the lack of financial incentive as an obstacle, in accordance with other research [10, 11]. Furthermore, patients and GPs may be unclear as to whether an additional appointment is needed after engagement with the FLS, with services split as to whether they recommended this or not. A previous UK GP survey also identified that GPs were not clear on whether or not further drug counselling was needed after FLS [11]. Taken together, these findings suggest some patients may get duplicate drug counselling, whilst others get none, and this is likely to contribute to the problem of poor adherence. Given that a core goal of FLSs is to promote drug uptake and adherence [12], and guidelines on medicines adherence emphasise the importance of involving patients in decisions about medicines [4], the degree to which FLSs engage with patients was an important characteristic to highlight in an adapted FLS typology.

Previous studies examining the characteristics of effective FLSs have focused on service setting, design, and intensity rather than the conduct or content of the consultation [13]. Our review of service pro formas is novel. Pro formas are often used by nursing, medical and allied health professionals to guide consultation content and have been shown to improve consultation quality, which has also been linked to patient safety [14]. Pro formas make the consultation and the process of data collection and data sharing more structured and may be helpful for training new staff less familiar with the clinical area, though may detract from a person-centred approach, and nurses have reported wanting to ‘know more’ to understand the biographical characteristics of a person’s situation [15]. Our observation of the pro formas was that the majority took a rather biomedical approach to risk factors and treatments, and perhaps missed an opportunity for a more holistic assessment. This is further relevant because a person’s personal situation and social circumstances might practically affect their ability to adhere to therapy, and adherence is known to be worse in osteoporosis than many other long-term conditions [16]. Our findings also demonstrate that pro formas often did not prompt the healthcare professional to check patient and/or carer understanding about where to access further information or support about osteoporosis. This is important because of the role that partners, family members and carers often play in supporting patient treatment decision-making and adherence [17, 18]. In addition, we observed that no service pro forma included reference to the discussion of pain or disability caused by fractures, which is recommended in UK FLS standards [1]. Our qualitative work identified that patient expectations of FLSs were incongruent with their experiences and it is important that clinicians align their agenda with that of the patient, who may be expecting the FLS to provide advice and support about their recent fracture. Furthermore, some pro formas missed opportunities to prompt balance, strength and mobility intervention, reflective of the FLS-DB national audit that identified only 6% of patients had started any such programme within 16 weeks of fracture in 2019 and 2020 [3].

A strength of this research is the multi-faceted approach including survey and pro forma review, both contributing to understanding consultation conduct and content. Limitations of this work include the survey response rate. Seventy-five FLSs in England and Wales submit data to the FLS-DB national audit [3]: we obtained information on 52% of services. To maintain anonymity of services contributing to this survey, we did not map our findings to the FLS-DB audit data [3]. This limits our ability to compare survey responses to service’s achievement of Key Performance Indicators. It is possible that service-related factors, not captured by this survey, may contribute to variation in consultation conduct and contact, such as how established the FLS is, changes in FLS structure or leadership, size of the FLS caseload, and the proportion of patients recommended drug treatment. Follow up and monitoring plays an important role to support osteoporosis medicine uptake and persistence [19]. Findings of this survey show that most services reported conducting 3–4-month follow-ups. This contrasts the FLS-DB national audit data, which demonstrates national variation in delivering follow ups and fewer than half of patients followed up within 16 weeks of their fracture [3]. This may suggest responder bias with participants reporting the best-case scenario. With respect to the pro forma analysis, the distinction between ‘partially’ and ‘fully’ met was subjective. The sample of pro formas was relatively small. For the services which provided a pro forma (*n* = 8), appointment duration varied from 20 to 50 min which would impact on the amount of content they could include in a consultation and pro formas, presumably, reflected this. Furthermore, a pro forma is a guide or aide memoire. Experienced clinicians may include other elements in their consultation which are not necessarily documented on these forms; therefore, we cannot assume that an absence on a pro forma correlates with absence of discussion in consultations.

A further limitation is that we used the UK ROS clinical standards [1] with which to map pro forma content, which may not necessarily reflect what should ideally be discussed in consultations or be relevant to international contexts. However, the ROS clinical standards are very similar in content to the International Capture the Fracture standards. Our previous consensus work, describing a model consultation has described important details of a FLS consultation [20] which are not reflected in current UK or international standards, including the need to ask about patient beliefs and thoughts about osteoporosis and osteoporosis medicines, to be positive, and to explain osteoporosis causes, consequences, and controllability, in order to increase perceptions of treatment need [20]. Further important elements might include exploring psychosocial elements such as fear of future falls or fracture, social withdrawal and cognitive impairment.

Our findings have helped inform the development of a decision support tool (iFraP) and the design of a trial to test the tool. We consulted with public and patients and our wider stakeholder group about how to adapt a decision support tool, that had originally been conceptualised for face-to-face consultations, for remote use. Although there was some support for patients being able to access an online tool at the same time as speaking to their clinician on the phone, our patient group felt this would involve too much technical and cognitive burden. We therefore decided that the online decision tool would simply be used by clinicians as a discussion aid when consulting by phone. The patient would then be sent a printout of the main information on the tool following the consultation, which would also include link to a website with videos of the tool being used so that they could see relevant images and animations. In terms of the trial, we used the findings to identify appropriate sites that both made decisions about, and discussed medicines with patients.

This research has clear implications for clinical practice. Firstly, we recommend that services use pro formas which reflect relevant clinical standards for FLSs. To support this, we have produced a ‘model’ pro forma based on the best components of submitted documents and the findings of this survey, available at https://doi.org/10.21252/r2bw-wz79. We encourage services to take a holistic, biopsychosocial approach to patient care which includes inquiry of symptoms and functional impairment relating to the index fracture. Second, in view of this study and previous research reiterating the uncertainty in roles between primary and secondary care in this clinical area, we suggest there is an urgent need to clarify professional roles. To do this, FLSs should ensure that their practices are clearly communicated to their local primary care and professional roles are clarified in the next iteration of UK and international clinical standards. Previous research has suggested that multi-faceted FLS interventions are associated with greater rates of treatment uptake [13]. We suggest that further study is given to the association between medication adherence and the amount of FLS direct patient contact to discuss medication.

## Conclusion

In summary, we have conducted the first survey of FLSs’ consultation conduct and content which identified large variation in who makes clinical decisions, whether medicines are discussed with patients or not, and in where and how prescribing takes place. A document analysis of FLS pro formas showed that these contained more content related to ‘investigating’ and ‘intervening’, rather than to ‘informing’. We have used the findings to expand on an existing FLS typology, and to design a new model FLS pro forma. Further research is needed to explore the relationship between FLS patient-practitioner contact and medicines adherence.

## Data Availability

Not applicable.

## Declarations

### Disclaimer

This study presents independent research funded by the NIHR. The views expressed are those of the author(s) and not necessarily those of the National Health Service, the NIHR, or the Department of Health and Social Care.
